# Phenolics Distribution in Rice and Their Macromolecular Interactions: A Matrix-Centric Perspective

**DOI:** 10.3390/foods15040660

**Published:** 2026-02-11

**Authors:** Halah Aalim, Muhammad Arslan, Hamza M. A. Abaker, Sulafa B. H. Hashim, Haroon Elrasheid Tahir, Naymul Karim, Mohammad Rezaul Islam Shishir, Xiaodong Zhai, Zhihua Li, Chenguang Zhou, Xiaobo Zou

**Affiliations:** 1 Agricultural Product Processing and Storage Lab, School of Food and Biological Engineering, Jiangsu University, Zhenjiang 212013, China; a.halah@outlook.com (H.A.);; 2 China Light Industry Key Laboratory of Food Intelligent Detection & Processing, School of Food and Biological Engineering, Jiangsu University, Zhenjiang 212013, China

**Keywords:** rice, polyphenols, matrix–phenolic interactions, binary and ternary systems, bioaccessibility, technofunctional properties, functional foods

## Abstract

Rice is a globally indispensable staple food and a major dietary source of phenolic compounds, whose nutritional and functional properties are influenced by their interactions within the rice matrix. This review provides a comprehensive synthesis of current knowledge on rice phenolics distribution and their macromolecule interactions, integrating evidence from binary, and ternary systems, to whole-matrix perspectives and examines their structural, functional, and nutritional consequences. Across rice genotypes, 76 polyphenols have been identified and quantified, encompassing phenolic acids, flavonoids, proanthocyanidins, and anthocyanins. Their abundance, chemical structure, and localization significantly dictated by grain anatomy, pigmentation, and processing. Mechanistically, phenolic binding is dominated by non-covalent interactions, including hydrogen bonding, hydrophobic interactions, electrostatic forces, CH–π interactions, and π–π stacking, facilitating multiscale structural reorganizations through amylose inclusion complexation, protein conformational rearrangements, lipid-assisted V-type crystallization, and dietary fiber binding. In ternary systems, competitive and synergistic interactions further modulate binding strength and structural organization. Functionally, these matrix-mediated interactions regulate stability and bioaccessibility of phenolic, macronutrient digestibility, glycemic response, and key technofunctional properties. By integrating compositional, mechanistic, and functional evidence, this review establishes a robust framework for understanding rice matrix–phenolic interactions and supports the rational design of phenolic-enriched, low-glycemic rice products with targeted nutritional benefits.

## 1. Introduction

Rice (*Oryza sativa* L.) is a principal cereal crop within the *Poaceae* family, tribe *Oryzoidae*, and constitutes a vital source of global nutrition and energy homeostasis, particularly in Asian countries. Nutritionally, rice is a rich source of carbohydrates and provides significant amounts of protein as well as essential micronutrients, including phosphorus, potassium, and B-complex vitamins [1]. Research has demonstrated that the intake of whole-grain and pigmented rice varieties is linked to a decreased risk of developing type 2 diabetes and other chronic diseases, such as obesity, hypertension, cardiovascular disorders and enhancements in gut microbiota [2,3,4]. These protective effects are predominantly ascribed to the grain’s bioactive secondary metabolites and their interactions with the rice matrix during cooking and processing [5,6]. Whole rice grains are processed into various products to enhance their taste and texture through both traditional methods, such as milling, cooking, and drying, as well as advanced techniques, including germination, fermentation, extrusion, and high-hydrostatic pressure processing, significantly influencing heat- and water-sensitive bioactive compounds [5,6,7]. These modulations positions rice as a sophisticated medium for delivering functional phytonutrients that contribute to sustained population health.

Phenolic compounds in rice represent a class of highly valuable bioactive constituents with strong potential for nutraceutical and functional food applications. Extensive research has demonstrated that phenolic compounds in rice and rice products exhibited a wide spectrum of biological activities, including suppress oxidative stress, anticancer, anti-obesity, anti-inflammatory, anti-aging, antidiabetic, anti-atherosclerotic, and neuroprotective effects [5,6,8,9,10,11]. The health-promoting potential of rice phenolics is governed by a complex interplay between intrinsic factors including genetic determinants and extrinsic factors, including extraction, purification, processing and matrix modifications, which together shape phenolic composition, and thermal stability [5,7,12,13,14,15,16,17,18]. Consequently, the functional and biological efficacy of rice phenolics exhibits significant variation across different rice products.

The physiological fate of rice phenolics is strongly dependent on their binding form. Free phenolics are primarily bioavailable in the upper gastrointestinal tract, whereas bound phenolics remain sequestered within the rice matrix until they undergo colonic fermentation, where microbial enzymes liberate them to generate bioactive metabolites with systemic effects [5,19,20]. Comprehending the distribution, binding forms, and processing implications on matrix interactions of rice phenolic compounds and matrix characteristics is essential for accurately predicting their functional efficacy and health implications [21,22,23,24]. Further, the literature on food-grade encapsulation systems shows that phenolic–matrix interactions are essential for determining phenolic stability, retention, controlled release, and ultimately bioaccessibility and functional performance in real food systems [25,26,27,28]. In rice, matrix–phenolic interactions have been linked to changes in starch structure and digestibility [29,30,31]. This understanding provides a scientific basis for the development of rice-based functional foods and nutraceuticals that deliver targeted health benefits.

Phenolic compounds in rice have been extensively investigated due to their structural diversity and recognized health-promoting potentials. However, the current literature remains fragmented, as most existing reviews primarily emphasize phenolic identification, compositional profiling, antioxidant capacity, or generalized bioactivity [32,33,34,35]. Furthermore, an increasing body of literature has investigated the behavior of phenolic compounds within simplified binary systems, typically involving starch or protein matrices [36,37]. However, these studies often fail to adequately address the mechanisms that govern phenolic functionality within the intact, multicomponent matrix. This reductionist approach limits mechanistic interpretation and the translation of the findings to real food systems. To address this critical gap, the present review synthesizes current evidence on rice phenolic composition, matrix components interaction, structural modulation, and associated functional outcomes within rice matrices. By adopting a matrix-centric and mechanistic perspective, this review provides a cohesive framework for advancing the understanding of the nutritional and metabolic effects of rice phenolics. It also supports the rational design of low-glycemic, phenolic-enriched rice-based foods and nutraceuticals aimed at enhancing metabolic health.

## 2. Review Methodology

This review critically examines and synthesizes research evidence on the phenolic composition of whole rice grains and their interactions within the rice matrix. The synthesis aimed to develop a matrix-centric framework that elucidates the distribution of phenolic compounds in rice and their interactions with major macromolecular constituents, including starch, proteins, lipids, and polysaccharides. Emphasis was placed on elucidating the impact of macromolecular interactions on phenolic localization, binding mechanism, matrix structure and functional outcome. A comprehensive literature search was conducted using the Scopus, Google Scholar, ScienceDirect, and Web of Science databases, covering English language peer-reviewed publications available up to December 2025. Searches were refined using Boolean operators as appropriate for each database. The search strategy employed a combination of keywords pertinent to rice varieties, phenolic classifications, and matrix interactions including: rice, *Oryza sativa* L., brown rice, pigmented rice, black rice, red rice, purple rice, wild rice, *Zizania aquatica* L., whole grains, starch, protein, lipids, polysaccharides, phenolic compounds, polyphenols, phenolic acids, flavonoids, anthocyanins, proanthocyanidins, matrix-phenolic interactions, starch–phenolic complex, protein–phenolic interaction, lipid–phenolic interaction, polysaccharide–phenolic interaction, starch–protein–phenolic interaction, starch–lipid–phenolic interaction, binary systems, ternary systems, amylose complexation, food matrix, macromolecular interactions, complex formation, and phenolic profile. Study selection prioritized publications providing mechanistic insight, structural characterization, or physicochemical evidence relevant to phenolic–macromolecule interactions, as well as studies addressing the comprehensive composition and spatial distribution of phenolics within whole rice grain tissues. Reference lists of selected articles were additionally screened to identify relevant studies not captured in the initial database search.

## 3. Overview of Phenolic Compounds in Rice

**Figure 1:**
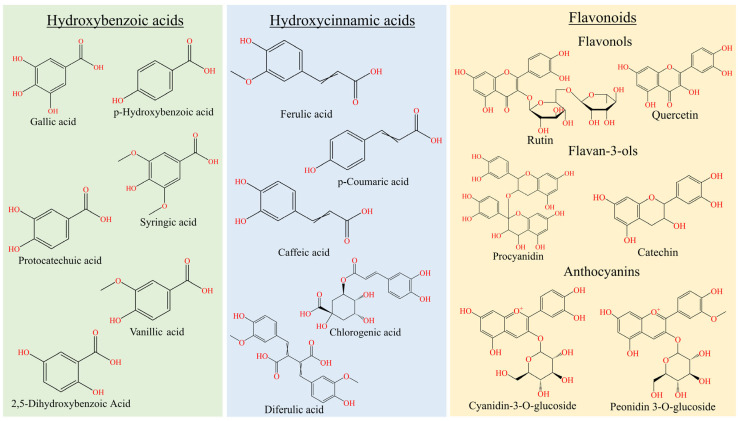
Schematic overview of the major classes of phenolic compounds presents within the rice.

Whole rice grain is produced by removing the husk while preserving the bran layers (pericarp, testa, and aleurone), endosperm, and embryo, which collectively determine the grain’s nutritional composition [38,39]. The fractions were characterized by the presence of varying quantities of phenolic profiles, starch, soluble sugars, proteins, and minerals [40,41,42]. Rice’s major phenolic groups are primarily classified as phenolic acids, and flavonoids which includes anthocyanins, and proanthocyanidins. These compounds are biosynthesized through the phenylpropanoid pathway, in which 4-coumarate serves as a central precursor that deviates into hydroxybenzoic acids, hydroxycinnamic acids, flavonoids, anthocyanins, and ultimately proanthocyanidins [34]. The biosynthesis of rice pigments is genetically regulated through loci such as *OsRc*, which are closely associated with total phenolic contents (TPC) and antioxidant capacity [43]. Comprehensive profiling has identified approximately 76 phenolic compounds in whole rice grains, as outlined in Table 1. Figure 1 provides a depiction of the primary rice phenolic constituents.

Rice phenolic compounds are present in three primary chemical forms: soluble-free, soluble-conjugated, and insoluble-bound fractions, indicating their biochemical functions and spatial distribution within the grain [44]. Insoluble-bound phenolics are covalently associated with cell wall components such as cellulose, hemicellulose, lignin, pectin, and structural proteins, whereas soluble phenolics are mainly stored in vacuoles [45,46]. Pigmented rice varieties, including black, red, and wild rice (*Zizania aquatica* L.), are characterized by a predominance of soluble-free phenolics, which account for 59–94% of TPC [47,48,49,50]. In contrast, non-pigmented brown rice contains mainly insoluble-bound phenolic acids, representing up to 89% of TPC [51]. Consequently, black and red rice exhibit substantially higher phenolic contents, with TPC values approximately 8.5-fold and 2.5-fold greater, respectively, than those of brown rice [52].

At the tissue level, rice bran represents the primary reservoir of phenolic compounds. Although it accounts for only 3.57–13.19% of grain weight, rice bran contains 60–97% of total grain TPC, depending on genotype [41,53]. Notably, the bran fraction contributes approximately 97% of total anthocyanin content (TAC) in whole grain [41]. The embryo, despite representing only 1.94–2.45% of grain weight, contributes 4–17% of TPC and approximately 3% of TAC [41]. Collectively, this distinct anatomical and chemical compartmentalization of phenolic compounds established the structural and biochemical basis for their interactions with starch, proteins, lipids, and dietary fiber. This, in turn, influences phenolic bioaccessibility, processing responsiveness, and functional outcomes within whole-grain rice systems.

**Table 1:** Summary of phenolic compound contents identified in various genotypes of whole rice grains (mg/100 g, dw).

#	Subclass/Compounds	Brown Rice	*n*	Red Rice	*n*	Black Rice	*n*	Wild Rice	*n*	References
	Hydroxybenzoic acids									
1	Gallic acid	TR-6.08	8	0.089–1.38	9	0.093–42.72	8	6.46–17.25	10	[6,14,48,50,54,55,56,57,58]
2	p-hydroxybenzoic acid	0.08–4.34	4	0.50	1	1.211–15.38	14	0.41–5.442	19	[6,14,48,49,55,57,58,59]
3	p-hydroxybenzaldehyde	3.682	1	–		–		1.21–1.56	2	[57,59]
4	2,5-dihydroxybenzoic acid	–		–		14.7	1	–		[55]
5	Ellagic acid	–		–		–		1.95–9.02	8	[48]
6	Vanillic acid	0.106–23.1	9	0.085–2.37	8	1.070–148.5	22	0.17–7.245	19	[6,14,48,49,50,54,55,56,57,59]
7	Isovanillic acid	0.46	1	–		–		–		[58,59]
8	Vanillin	5.784	1	0.1	1	–		1.3–2.23	2	[49,57,59]
9	Syringic acid	0.09–2.499	9	0.100–2.52	8	0.080–14.18	8	0.18–4.589	19	[6,14,48,49,50,54,55,56,57,59]
10	Syringaldehyde	3.86	1	1.38	1	6.76	1	–		[6,14]
11	Protocatechuic acid	0.086–1.21	6	0.104–14.12	10	1.28–143.23	10	0.77–3.31	10	[6,48,50,54,55,57,59,60,61]
12	Protocatechuic aldehyde	1.266	1	–		–		–		[14,59]
13	Protocatechuic acid ethyl acid	–		–		–		0.05–0.61	10	[48,57]
14	α-Resorcylic acid	1.36	1	–		–		–		[58]
15	Gentisic acid	1.23	1	–		–		–		[58]
16	Salicylic acid	2.65	1	–		–		–		[58]
	Hydroxycinnamic acids									
17	Ferulic acid	0.055–49.43	11	0.037–32.21	10	1.04–251.35	20	12.11–40.644	19	[6,14,48,49,50,54,55,56,57,58,59,60,62]
18	Diferulic acid	1.637–1918.8	2	2445.5	1	2332.9	1	2.878–3.358	9	[49,60]
19	8-*O*-4-Diferulic acid	1.06	1	–		–		–		[49]
20	8-5- benzofuran diferulic acid	1.549	1	–		–		–		[49]
21	Triferulic acid	1120.3	1	879.2	1	894.6	1	–		[60]
22	Ferulic acid *O*-dihexoside *	988.34	1	220.58	1	713.79	1	–		[60]
23	Isoferulic acid	1.379–8.895	3	0.35	1	12.19–26.74	2	–		[6,14,49,55,59]
24	*p*-coumaric acid	ND–9.701	10	ND–6.55	10	ND–20.99	21	0.23–8.46	19	[6,14,48,49,50,54,55,56,57,58,59,60]
25	O-Coumaric acid	0.087–0.191	4	0.14–0.896	7	0.27–0.93	6	0.22–1.0	10	[48,50]
26	M-Coumaric acid	11.08–2.362	2	–		17.51	1	–		[49,60]
27	Sinapic acid	0.785–2.8	4	1.61–0.32	2	27.22	1	2.68–9.87	19	[6,48,49,57,59,60]
28	cis-Sinapic acid	1.259	1	–		–		–		[49]
29	Disinapic acid	–		–		–		1.015–1.878	9	[49]
30	Sinapic acid *O*-dihexoside *	27.1		22.22	1	57.9		–		[60]
31	Chlorogenic acid	1.775–3.32	4	2.12	1	11.26	1	0.92–1.92	8	[6,14,48,49,56,59]
32	Caffeic acid	0.07–4.677	4	0.56–2.68	5	0.27–1.85	18	0.16–1.11	8	[6,14,48,49,50,56,58,59]
33	cis-caffeic acid	0.017	1	–		–		–		[49]
34	Cinnamic acid	–		0.09–0.18	4	0.1–3.06	6	0.58–1.87	8	[6,48,50,52]
35	3-p-Coumaroylquinic	0.072	1	0.128–0.131	2	0.049–0.7	3	–		[52]
36	3-Feruloylquinic	0.056	1	0.068–0.101	2	0.091–1.21	3	–		[52]
37	4-p-Coumaroylquinic	–		0–0.007	2	0.012–0.141	3	–		[52]
38	4-Feruloylquinic	–		0–0.031	2	0.01–0.226	3	–		[52]
	Flavonols									
39	Quercetin	TR	1	0.25–3.14	6	0.19–4.29	21	1.01–4.41	10	[6,14,48,50,52,57,58]
40	Quercetin-3-*O*-glucoside (isoquercetin)	0.379	1	0.102–0.166	2	0.15–4.8	17	–		[52,58,60,63]
41	Dihydroquercetin-3-glucoside	0.043	1	0.034–0.045	2	0.06–0.806	3	–		[52]
42	Rutin (Quercetin-3-*O*-rutinoside)	1.57	1	0.24–2.08	4	0–4.32	22	8.36–37.49	10	[6,48,49,50,52,57,58,63]
43	Kaempferol	0.39	1	2.72	1	0.04–1.23	13	0.91–1.37	8	[6,14,48,58]
44	Isorhamnetin	0.001–1.40	2	0.007–2.09	3	0.122–2.51	4	–		[6,14,52]
45	Isorhamnetin 3-*O*-glucoside	0.012	1	0.006–0.085	2	0.177–0.6	5	–		[52,60,63]
46	Isorhamnetin-3-*O*-rutinoside	–		–		0.006–0.127	3	–		[52]
47	Dihydroisorhamnetin-3-*O*-glucoside	0.04	1	0.012–0.018	2	0.052–0.124	3	–		[52]
	Flavons									
48	Luteolin-6,8-C-pentoside-6,8-C-hexoside *	–		–		0.5–1.0	1	–		[63]
49	Apigenin-6,8-C-pentoside-8,6-C-hexoside *	–		–		1.4–4.8	1	–		[63]
	Flavan-3-ols									
50	Catechin	0.103–0.209	4	0.414–5.32	10	0.029–7.89	3	1.56–3.45	10	[48,50,52,54,57,60]
51	Epicatechin	3.76	1	0.35–7.61	2	6.35	1	2.43–12.32	10	[6,14,48,49,57]
52	Epigallocatechin	–		0.142–0.244	2	ND–0.308	3	0.75–7.93	10	[48,52,57]
53	Procyanidin dimer B1	–		0.7–4.09	4	ND–9.02	3	1.02–1.3	2	[52,57,60]
54	Procyanidin dimer B2	–		0.24–0.794	3	ND–1.52	3	0.5–0.55	2	[52,57]
55	Procyanidin dimer B3	–		0.34	1	–		0.63–0.94	2	[57]
56	Procyanidin dimer B4	–		0.235–0.352	2	ND–0.576	3	–		[52]
57	Procyanidin dimer C2	–		0.6	1	–		1.7–2.42	2	[57]
58	Oligomers	–		52.5–84.4	2	5.33–155.8	3	–		[52]
	Flavanons									
59	Hesperetin	–		–		ND–0.81	12	–		[58]
60	Taxifolin *O*-hexoside *	–		–		11.87	1	–		[60]
	Anthocyanins									
61	Cyanidin-3-*O*-glucoside	–		–		1.2–395.267	36	0.3	1	[6,41,44,47,52,54,55,58,60,61,63,64,65]
62	Cyanidin-3,5-*O*-diglucoside	–		–		1.3–1.86	2	–		[60,63]
63	Cyanidin 3-*O*-rutinoside	–		–		0.44–6.33	15	–		[41,52,60,65]
64	Cyanidin-3-*O*-arabidoside	–		–		0.3	1	–		[63]
65	Cyanidin-3-*O*-gentiobioside	–		–		3.79–4.20	3	–		[52]
66	Cyanidin-3-*O*-(6″-*O*-p-coumaryl)glucoside	–		–		0.4	1	–		[63]
67	Epicatechin/catechin-cyanidin-3-glucoside	–		–		3.85–6.54	3	–		[52]
68	Peonidin 3-*O*-glucoside	–		–		0.35–31.1	25	–		[41,44,47,52,54,55,58,60,61,63,64]
69	Peonidin 3-*O*-rutinoside	–		–		0.13–3.69	4	–		[52,60]
70	Epicatechin/catechin-peonidin-3-glucoside	–		–		3.25–3.52	3	–		[52]
71	Peonidin-3-*O*-(6″-O-p-coumaryl)glucoside	–		–		0.3	1	–		[63]
72	Malvidin	–		–		0–0.319	10	–		[65]
73	Malvidin-3-*O*-glucoside	–		–		3.21–3.34	3	–		[52]
74	Pelargonidin-3-*O*-glucoside	–		–		0.6–48.746	11	–		[63,65]
75	Delphinidin	–		–		0.137–8.699	10	–		[65]
76	Delphinidin-3-*O*-glucoside	–		–		0.285–1.51	10	–		[65]

#, compound number; *, hexose may refer to glucoside or galactoside; *n*, number of samples; dw, dry weight; ND, not detected; TR, trace amount (<LOQ); – not reported.

### 3.1. Phenolic Acids

Phenolic acids abundance, chemical form, and spatial localization underpin many of the structural and functional attributes of rice phenolics. Phenolic acids are the dominant class of phenolic compounds widely distributed in rice grains, occurring primarily as hydroxybenzoic acids and hydroxycinnamic acids [32]. Across rice genotypes, insoluble-bound phenolic acids represent the major fraction and showed pronounce variation among different varieties [44]. At the cellular level, the distribution of phenolic acids was markedly heterogeneous, indicating a differential association with specific grain tissues and subcellular compartments [50]. For instance, ferulic and *p*-coumaric acids consistently emerged as the predominant insoluble-bound phenolic acids in all three *Oryza* genotypes, where they are ester- or ether-linked to cell wall polysaccharides and lignin [44,51,60]. Additional contributors to the insoluble-bound pool included sinapic acid, as well as di- and tri-ferulic acids, which participated in cell wall crosslinking [60]. In contrast, m-coumaric and o-coumaric acids have been detected in both soluble-free and insoluble-bound fractions across *Oryza* genotypes [50,54,60].

Genotype and pigmentation-dependent differences further shaped phenolic acid profiles. In pigmented rice, protocatechuic acid predominates in the soluble-free fraction, was absent in brown rice varieties in all phenolic forms, together with 2,5-dihydroxybenzoic acid [44,60]. Conversely, vanillin has been consistently reported in brown, milled, and basmati rice, where it contributed significantly to aroma development [59]. Additionally, hydroxycinnamoylquinic acid derivatives, including 3- and 4-*p*-coumaroylquinic and feruloylquinic acids, were present across rice genotypes, with feruloylquinic acids dominating in black rice and p-coumaroylquinic acids enriched in red rice [52]. Wild rice exhibited a distinct phenolic acid spectrum, dominated by ferulic, gallic, sinapic, protocatechuic, and ellagic acids [48,49]. This compositional heterogeneity is central for understanding phenolic–matrix interactions, extractability, and phenolic acids behavior during rice processing and digestion.

### 3.2. Flavonoids

Flavonoids are predominantly localized in the bran layer of the rice caryopsis, and their accumulation has been positively associated with grain morphology, particularly increased kernel length and length-to-width ratio [66]. Consistent with trends observed for TPC, total flavonoid content (TFC) is significantly higher in pigmented rice varieties than in non-pigmented counterparts [6,14,44,67]. Rice flavonoids encompass several subclasses, including flavonols, flavones, flavanols (flavan-3-ols), flavanones, isoflavones, and anthocyanins [32].

In pigmented rice, flavonoids occur predominantly in the soluble-free fraction, accounting for approximately 74–96% of total flavonoids, although appreciable proportions of insoluble-bound flavonoids (4–50%) have also been reported [50,67]. Variety-dependent subclass distribution was observed as flavonols dominate the soluble-free fraction in black rice, and flavan-3-ols were the major free flavonoids in red rice [52,60]. Among flavonols, quercetin-3-*O*-glucoside was the predominant compound in brown rice, representing 94.4% of total flavonols, followed by red rice (88.1%), while black rice is characterized by a more diverse flavonol profile, including quercetin, quercetin-3-*O*-rutinoside, and isorhamnetin-3-*O*-rutinoside [50,52].

Minor flavonoids such as dihydroquercetin (taxifolin) and dihydroisorhamnetin have also been detected across different *Oryza* genotypes, albeit at low concentrations [52]. The occurrence of (+)-catechin is inconsistent in brown rice, whereas it represents the dominant flavan-3-ol monomer in red rice varieties, accompanied by lower levels of (−)-epicatechin, (−)-gallocatechin, and (−)-epigallocatechin [14,50,52,54]. Collectively, these genotype- and fraction-specific differences highlight the strong influence of pigmentation and grain structure on flavonoid composition, localization, and potential reactivity within the rice matrix.

#### 3.2.1. Anthocyanins

Anthocyanins are the major hydrophilic flavonoid constituents in pigmented rice and are predominantly localized within the vacuoles of the pericarp, where they are responsible for the characteristic red, purple, and black pigmentation of the grain. Their presence and abundance were strongly genotype-dependent, explaining the marked differences in pigmentation among rice varieties [6,52,63,65,68]. In pigmented rice grains, anthocyanins are detected almost exclusively in the soluble-free fraction, reflecting their vacuolar storage and high polarity [60,69]. Among pigmented rice types, black rice consistently exhibits the highest TAC, exceeding that of red and wild rice varieties [68,70]. Correlation analyses between color parameters and phenolic profiles further indicate that grain pigmentation is closely associated with the accumulation of soluble-conjugated phenolic precursors, including protocatechuic, vanillic, and *p*-coumaric acids, which are part of the anthocyanin biosynthetic pathway [44].

Across black and purple rice varieties, cyanidin-3-*O*-glucoside (C3G) was the dominant anthocyanin, accounting for approximately 60–89% of TAC [6,52,60,63,65,68,71,72,73]. Followed by secondary anthocyanins including peonidin-3-*O*-glucoside (7.6–15%), cyanidin-3,5-di-*O*-glucoside (5.5%), and cyanidin-3-*O*-rutinoside (4.2–4.5%) [52,60,61,63,73]. In addition, anthocyanins such as cyanidin-3-*O*-arabinoside, cyanidin-3-*O*-gentiobioside, delphinidin-3-*O*-glucoside, petunidin-3-*O*-glucoside, and malvidin-3-*O*-glucoside have been reported at trace levels [52,60,63,71,72]. Acylated derivatives, including cyanidin- and peonidin-3-*O*-(6″-*O*-p-coumaroyl)glucosides, as well as pelargonidin-based anthocyanins, have also been identified in black and purple Japanese rice varieties [63]. Furthermore, flavanol-anthocyanin direct adducts, such as (epi)catechin-cyanidin-3-glucoside and (epi)catechin-peonidin-3-glucoside, have been detected at trace levels in Italian hybrid and native rice varieties, indicating in situ condensation reactions within the grain matrix [52].

In red rice varieties, the occurrence of anthocyanins remains controversial. Several studies reported C3G as the predominant anthocyanin, contributing 30–67% of TAC [68,72], followed by peonidin-3-*O*-glucoside and cyanidin-3,5-di-*O*-glucoside [68]. However, other investigations have failed to detect anthocyanins in red rice varieties [14,44,52]. This apparent discrepancy was likely attributable to metabolic flux within the flavonoid pathway, as anthocyanidins serve as intermediates for proanthocyanidin biosynthesis, which dominates pigmentation in red rice [69]. Consequently, red rice pigmentation was primarily associated with condensed tannins rather than anthocyanin accumulation.

#### 3.2.2. Proanthocyanidins

Proanthocyanidins, also referred to as condensed tannins, are oligomeric and polymeric flavonoids composed primarily of (+)-catechin and (−)-epicatechin units. In rice grains, they are mainly stored in vacuoles of the caryopsis and are predominantly detected in the soluble-free fraction, consistent with their biosynthetic origin and intracellular localization [67,69,74,75]. Proanthocyanidins represent the principal phenolic class in red rice varieties, where they occur mainly as oligomers with degree of polymerization (DP) of 5–8, accounting for approximately 40% of total proanthocyanidins, followed by highly polymerized forms (DP > 10) contributing about 29% [14,44,67,69].

Structurally, rice-derived proanthocyanidins exhibited a broad molecular weight range (500–18,000 Da) and a wide span of polymerization (DP 1–38), with (+)-catechin acting as the dominant terminal and extension unit across pigmented rice varieties [52,75]. These polymers play a functional role in grain pigmentation, the oxidative polymerization of catechin dimers has been implicated in the development of the characteristic red color during grain ripening [76]. Specific procyanidin dimers, including procyanidin B1, B2, and B4, have been identified as abundant constituents in pigmented rice varieties [52].

Although brown rice varieties are frequently reported to lack detectable proanthocyanidins [14,44,54,69], trace to low levels have been quantified in certain American brown rice cultivars, reaching 6–9 mg (+)-catechin equivalents per 100 g grain [67], indicating genotype-dependent variation. Reports on black rice are similarly inconsistent. Some studies reported an absence of proanthocyanidins [44], whereas others have documented appreciable concentrations [6,52,67,69]. These discrepancies likely reflect differences in analytical methods, extraction solvents, degree of polymerization, and cultivar selection.

## 4. Rice Matrix–Phenolic Binary Interactions

### 4.1. Starch–Phenolic Interactions

**Figure 2:**
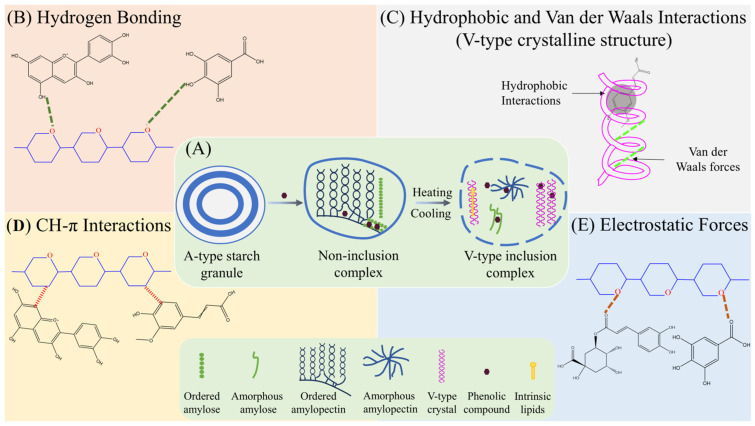
Schematic overview of the proposed multi-scale rice starch–phenolic interactions. (**A**) non-inclusion adsorption of phenolics onto starch granule surface and V-type crystalline structure inclusion complex, (**B**) hydrogen bonding, (**C**) hydrophobic and van der Waals interactions, (**D**) CH–π interactions, and (**E**) electrostatic forces.

Starch is the predominant macronutrient in rice, accounting for approximately up to 90% of grain dry weight [77,78]. It is composed of amylose and amylopectin organized into alternating crystalline and amorphous lamellae, together defining the hierarchical structure of the starch granule [78,79]. Owing to its abundance, intrinsic structural heterogeneity, and high susceptibility to thermal, mechanical, and enzymatic modification [80,81,82,83], starch represents the primary macromolecular partner for phenolic interactions within rice matrices. Table 2 summarizes the literature findings of rice starch and phenolic compounds interactions, and Figure 2 depicts the proposed interaction mechanisms.

The interaction between rice starch and phenolic compounds is primarily governed by non-covalent forces, specifically hydrogen bonding and hydrophobic interactions. These interactions are significantly influenced by the molecular architecture of the phenolic compounds, which ultimately determines the structural assembly and functional specificity of the resulting complexes. Multiscale mechanistic analyses by Chen et al., [87,88] demonstrated that polyhydroxylated acids such as gallic acid exhibit high-affinity hydrogen bonding with amylose helices, forming bridge-like macromolecular assemblies with strong binding energies (−3.17 kcal/mol), increased relative crystallinity (18–22%), and marked reductions in starch digestibility (31–35%), indicating significant structural stabilization. In contrast, methoxylated phenolics like syringic acid interact predominantly via hydrophobic associations within the helical cavities of amylose, yielding a lower binding energy (−2.86 kcal/mol) but still contributing to improved starch thermal resistance. Meanwhile, cinnamic acid derivatives (e.g., ferulic and caffeic acids) displayed dual behavior: their extended aromatic backbone allows partial penetration into hydrophobic cavities while simultaneously engaging in hydrogen-bond networks within amorphous regions, enabling both inclusion and surface adsorption interactions. These findings showed that phenolic molecular structure governs starch interaction mechanism, while binding site specificity determines the complexes’ functional behavior.

The primary factors determining the strength of starch–phenolic interactions are amylose content and accessibility, which impact the complexes inclusion mechanisms through V-type crystallinity, co-stabilization, or non-inclusion surface interactions [95,97,98,99,100]. Generally, processing modifies the physical properties of starch, which subsequently affects amylose availability, therefore influencing binding efficiency [101,102,103,104,105,106]. Gelatinized starch, which characterized by granule swelling and amylose leaching, exhibits the highest complexation capacity across diverse phenolic classes [89,92,93]. Retrograded and debranched starches further amplify complex formation by providing linear templates suitable for inclusion complexes, leading to enhanced resistant starch formation [86,88,107]. By contrast, native starch, where amylose remains largely confined within intact granules, exhibited limited interaction and weaker functional modification [90]. Thus, selecting amylose-rich matrices in optimal physical states is critical for tailoring targeted starch–phenolic systems.

Starch–phenolic interactions significantly modify the physicochemical, structural, and digestive behavior of rice starch systems, enabling deliberate modulation of nutritional and technological performance. A wide range of rice phenolics including gallic acid, ferulic acid, chlorogenic acid, catechins, quercetin, and crude rice phenolic extracts consistently demonstrated their ability to alter gelatinization behavior, retrogradation tendency, viscoelastic properties, and starch digestibility [84,85,87,88,90,108]. Experimental systems employing hydrothermal complexation, extrusion, and enzymatic debranching consistently demonstrated these functional improvements [85,88,89,90,105]. While non-thermal processing strategies such as ultrasound, high-pressure homogenization boosted amylose–phenolic integration, resulting in higher resistant starch yields compared to native starch [81,89,91,92]. Further, phenolics consistently inhibited starch retrogradation, shown by reduced enthalpy and ratio of the retrogradation [90,93,95], and lowered viscosity profiles [84,93,96], decreased setback, and altered gel firmness [90,93], syneresis [88], enhance phenolic antioxidant capacity [86,92], and function in food products [109,110]. Collectively, these structural and functional modifications position starch–phenolic complexes as versatile, clean-label tools for designing low-glycemic functional food, antioxidant-rich matrices, and retrogradation-resistant rice products.

### 4.2. Protein–Phenolic Interactions

**Figure 3:**
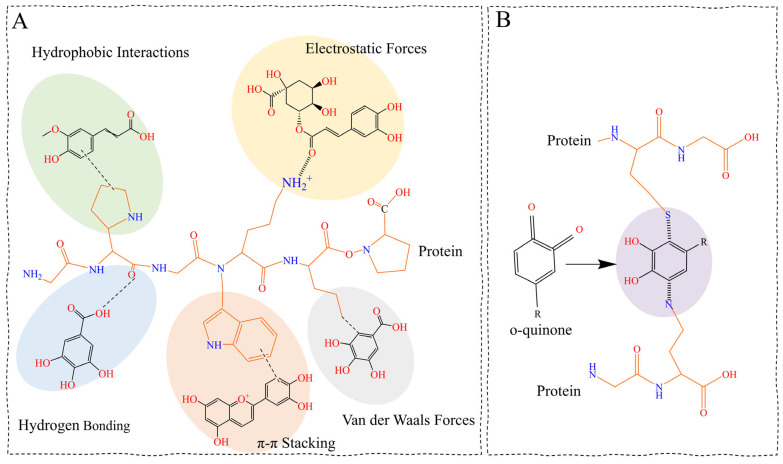
Mechanisms of rice protein–phenolic interactions. (**A**) Non-covalent interaction pathways involving hydrogen bonding, hydrophobic interactions, electrostatic interactions, van der Waals forces, and π–π stacking. (**B**) Covalent conjugation pathway involves phenolic oxidation to quinone intermediates, followed by C–N and C–S bond formation with nucleophilic amino acid residues in rice proteins.

Rice is a valuable source of plant proteins due to its relatively high digestibility, nutritional value, and extensive applications in the food industry, particularly in the production of gluten-free food products [111,112]. In rice, crude protein constitutes approximately 6–15% of the grain weight, with glutelin being the most abundant fraction accounting for about 70% of the total protein, followed by albumin, globulin, and prolamin [78]. Rice protein is a significant source of essential amino acids necessary for human health, including histidine, isoleucine, lysine, methionine, phenylalanine, valine, and threonine [78]. Table 3 summarizes the literature findings of rice protein and phenolic compounds interactions, and Figure 3 depicts interaction mechanisms.

Rice proteins bind phenolic compounds through either non-covalent and/or covalent interaction mechanisms, significantly impacting both protein functionality and the fate of phenolics [126,127]. Non-covalent interactions are reversible and are driven mainly by hydrogen bonding and hydrophobic forces, with additional contributions from van der Waals and electrostatic interactions, which have been documented using FTIR, fluorescence quenching, circular dichroism spectroscopy and molecular docking [119,122,123,125]. Non-covalent forces alter protein conformation without changing its primary structure and are sensitive to environmental conditions such as pH, ionic strength, and temperature [115,128]. Binding with gallic acid, epigallocatechin gallate, catechins, and anthocyanins was reported to reduces protein α-helix content and increases β-sheet or random coil structures [118,119,125]. These interactions disrupted the local hydrogen bonding and exposed buried hydrophobic residues, leading to partial unfolding or re-packing.

In contrast, covalent rice protein–phenolic interactions lead to permanent chemical modifications that induce greater and more persistent structural and functional changes. These reactions occur when phenolic compounds undergo enzymatic or chemical oxidation to form highly reactive quinone or phenoxyl radical intermediates (Figure 3B), which subsequently form new C–N or C–S bonds with nucleophilic amino acid side chains, primarily lysine, cysteine, and histidine, and in some cases tryptophan or tyrosine [15,115,121]. Covalent conjugation anchors phenolics into the protein backbone, generating more permanent conformational changes [114,115,116]. Covalent conjugation has been extensively documented in systems that involve the laccase- and oxidant-mediated linkage of polyphenols, such as ferulic acid, gallic acid, and tannic acid. This process results in stable protein–phenolic conjugates that exhibit significantly reduced sensitivity to moderate variations in pH, ionic strength, digestion, or processing conditions [15,115,121]. As a result, covalent phenolic conjugation serves as an effective mechanism for permanently modifying protein structure, stability, and functionality within the food matrix.

The molecular structure of phenolic compounds, including molecular size, polarity, the number and position of hydroxyl groups, and aromatic configuration, fundamentally determines their binding affinity, mode of interaction, and consequent functional outcomes. For instance, gallic acid primarily interacts through hydrogen bonding, resulting in only moderate structural modifications in proteins [125], whereas ferulic acid and procyanidin dimers exhibit stronger binding via hydrophobic interactions [120,122]. Catechins and epigallocatechin gallate exhibit strong dual-mode binding, combining hydrogen bonding and hydrophobic interactions, which is reflected in pronounced fluorescence quenching and conformational changes [119,123]. Tannic acid, owing to its high molecular weight and multivalent structure, promotes extensive cross-linking, leading to substantial reductions in protein digestibility [115]. Anthocyanins form their most stable complexes under acidic conditions, where electrostatic and π–π interactions are favored [118]. In general, phenolics with multiple hydroxyl groups preferentially form hydrogen bonds, while those with larger aromatic systems favor hydrophobic and π–π interactions [113,114,118]. Polymeric phenolics, characterized by multiple aromatic rings and abundant hydroxyl groups, enable multi-site binding and multipoint interactions with proteins, thereby amplifying their structural and functional changes [113,115,119,125]. Consequently, strategic selection of phenolic structures allows targeted modulation of protein conformation, solubility, antioxidant retention, and interfacial functionality.

Processing conditions modulate protein structure and phenolic reactivity, enabling predictable tuning of binding strength and complex stability. Mild heating enhances non-covalent complexation [124], while protein enzymatic hydrolysis increases peptide accessibility and improves phenolic grafting [121]. Laccase oxidation reliably produces covalent conjugates with controlled grafting degrees [15,115]. High hydrostatic pressure and ultrasound treatments accelerate unfolding and mass transfer, boosting both covalent and non-covalent interaction efficiency [115,117,118,129,130]. Protein unfolding process reveal hydrophobic and reactive sites, whereas phenolic oxidative treatments facilitate permanent covalent bonding [113,114,124,125,131,132]. Optimizing processing conditions is crucial for achieving desired functional outcomes.

Rice protein–phenolic interactions significantly enhance functional performance by improving protein solubility, emulsifying capacity, antioxidant stability, and phenolic digestive stability. Non-covalent complexes with gallic acid, and epigallocatechin gallate, showed the highest solubility and interfacial activity due to mild protein unfolding and reduced hydrophobic aggregation [119,123,125], whereas covalent conjugates with ferulic acid, chlorogenic acid and catechins can further improve or reduce solubility depending on cross-linking intensity [115,121]. These interactions consistently enhanced the emulsifying stability, thermal stability, and foaming [113,114,121,123], particularly at pH 3 for anthocyanin complexes or moderate catechin grafting levels [116]. They also strengthen oxidative stability, with protein–phenolic films reducing peroxide and TBARS formation in emulsions [116,121,122]. Importantly, rice protein matrices act as delivery systems, stabilizing phenolics during digestion and improving intestinal recovery [15,115,124]. These advantages emerge due to the reorganization of protein conformation facilitated by phenolic binding, which enhances interfacial adsorption and modulates protein function [121,125,133]. Thus, rice protein–phenolic complexes provide versatile, clean-label strategies to improve stability, antioxidant function, and bioactive delivery in modern food systems.

## 5. Binary Interactions in Rice Matrix and Their Role in Phenolic Binding

### 5.1. Starch–Protein Binary Interactions

Starch–protein interactions represent a fundamental physicochemical mechanism that governs starch structural reorganization, digestion kinetics, and the matrix environment in which phenolic compounds are retained and functionally expressed in rice [134,135,136]. At the molecular level, starch–protein interactions in rice are predominantly driven by spontaneous non-covalent binding, primarily hydrogen bonding and hydrophobic interactions [137,138,139]. Further, native and enzymatically hydrolyzed rice proteins have been shown to promote ordered starch structures, and facilitate V-type inclusion complex formation [140,141,142]. Protein coatings and starch–protein aggregates act as physical barriers that restrict starch swelling, granule disintegration, suppress starch retrogradation, and enzyme access to hydrolysis sites [80,136,139,140,143,144,145,146,147]. Further, protein hydrolysis degree and pH were found to be powerful regulators of starch–protein interactions, directly influencing texture, water distribution, and matrix stability [148]. Spectroscopic and structural analyses showed that amylose–glutelin binding induces pronounced protein conformational rearrangements, including increased α-helix content, reduced β-sheet structures, decreased surface hydrophobicity, and preferential interactions with protein [149,150]. Such molecular-level order explains the alteration in starch digestion behavior in protein-rich rice systems.

The interactions among starch, protein, and phenolic compounds within a ternary system result in emergent behaviors that cannot be predicted solely from the examination of binary interactions. Anthocyanins have been shown to interact concurrently with starch and protein, inducing composite structural rearrangements that reduce enzymatic accessibility of both macromolecules [36]. Variations in protein content further modulate the functional outcomes of these composite systems, influencing phenolic binding strength and digestion behavior [137,139]. In rice starch–protein–C3G system, increasing protein levels to 10% enhanced C3G binding, reduced the particle size and boosted resistant starch formation [139]. Conversely, anthocyanins from pigmented rice induced protein conformational rearrangements that increased proteolysis resistance while simultaneously reducing α-amylase accessibility through starch complexation [36]. Such alterations limit enzyme access and phenolic release during digestion [16,139]. Together, these findings establish ternary rice starch–protein–phenolic interactions as a unifying mechanistic framework explaining the non-linear effects observed on starch digestibility, protein utilization, and phenolic bioavailability in rice-based foods.

### 5.2. Starch–Lipids Binary Interactions

Starch–lipid interactions in rice constitute a structurally decisive mechanism that reorganizes amylose architecture and underpins key physicochemical and nutritional properties of rice-based systems. Lipidomic analyses further reveal that starch granule-associated lipids are distributed both on granule surfaces and within internal lamellae and dynamically accumulate during grain filling, highlighting their intrinsic role in starch biosynthesis and hierarchical organization [151]. The presence or removal of lipids significantly affects starch retrogradation, thermal transitions, and rheological properties, particularly in cooked and stored rice [152,153]. At the mechanistic level, the complexation of amylose with lipids, commonly referred to as V-type complex formation, creates hydrophobic binding domains within single-helical amylose structures [154,155,156]. This reorganization alters the crystalline–amorphous balance, restricts molecular mobility, and suppresses starch swelling and retrogradation, while promoting dense, enzyme-resistant configurations. As a result, starch–lipid complexes contribute to increased resistant starch formation and reduced enzymatic accessibility [157]. These effects establish starch–lipid interactions as foundational architectural modifications that directly govern digestion resistance and controlled glucose release in rice matrices.

Processing interventions further strengthen starch–lipid interactions and amplify their impact across multiple structural scales. Hydrothermal, cold plasma, and ultrasound treatments enhanced lipid-bound amylose content and reorganized the starch matrix, even without substantially increasing total lipid levels, leading to reduced starch digestion [16,154,155,158,159]. Structural analyses consistently reported multiscale reorganization, encompassing V-type helices, compact starch granules, and reinforced gel networks characterized by altered crystallinity, pore architecture, and water mobility [160,161]. These coordinated structural changes restrict water diffusion and enzyme penetration, reinforcing digestion resistance from the molecular to the macroscopic gel level [152]. Such processing-enabled control over starch–lipid architecture provides a mechanistic basis for tailoring rice texture, shelf stability, and metabolic response.

Starch–lipid interactions markedly enhance phenolic binding by enabling the formation of stable ternary amylose–lipid–phenolic complexes that further reorganize starch structure and modulate nutritional functionality. Recent studies demonstrate that the synergistic presence of rice bran oil, fatty acids (e.g., stearic acid) and phenolic compounds (e.g., quercetin or gallic acid) promotes the formation of V-type crystalline structures classified as resistant starch type 5 (RS5) [157,162]. Lipid-induced hydrophobic domains stabilize amylose single helices by accommodating lipid molecules within the helical cavity, while phenolics bound to the helix exterior via hydrogen bonding and hydrophobic interactions, collectively enhancing phenolic retention and modulating their release during digestion [163]. This cooperative assembly produces dense molecular configurations, with relative crystallinity reported to increase dramatically (from 11.6% to 41.4%) in model rice starch systems [162].

The molecular structure of phenolic compounds plays a critical role in determining the stability of complexes. Specifically, phenolics such as quercetin and gallic acid enhance the inclusion of amylose–lipid complexes, while larger flavonoids like rutin may impede complex formation at initial stages due to steric hindrance [162,164]. Importantly, these ternary complexes sequester phenolics, protecting them from thermal and oxidative degradation while simultaneously restricting enzymatic access to starch [157,158,162,165]. By coupling phenolic stabilization with reduced starch digestibility, starch–lipid–phenolic interactions emerge as a powerful molecular strategy for designing low-glycemic, phenolic-enriched rice-based foods with enhanced metabolic function.

## 6. Role of Non-Starch Polysaccharides in Phenolic Binding and Matrix Function

Non-starch polysaccharides (NSPs) in rice act as the principal reservoirs for bound phenolic compounds and are the primary regulators of phenolic stability, bioaccessibility, and site-specific release during digestion. Phenolic compounds, particularly hydroxycinnamic acids such as ferulic and *p*-coumaric acids are predominantly present in bound forms within rice bran and outer grain layers, where they are covalently linked to NSPs including cellulose, arabinoxylans, and hemicelluloses [166,167]. In vitro digestion studies consistently showed minimal phenolic liberation during gastric and small-intestinal phases (2.68%), whereas colonic fermentation results in substantially higher release (27.57%), accompanied by increased antioxidant capacity, α-glucosidase inhibition, and prebiotic effects [168]. The dense, cross-linked NSPs architecture restricts enzymatic accessibility in the upper gastrointestinal tract, effectively protecting phenolics from premature degradation [169], while microbial enzymes such as xylanases, esterases, and cellulases depolymerize NSPs networks in the colon, enabling phenolic release and biotransformation into bioactive metabolites [5,170]. As a result, phenolic bioavailability is shifted toward the gut, where phenolic metabolites exert antioxidant, metabolic, and microbiota-modulating effects.

Beyond intrinsic phenolic esterification, NSPs can assemble into a composite structural matrix that integrates interactions with starch, proteins, lipids, and water that govern phenolic binding and release. NSPs such as cellulose, hemicellulose, and functional hydrocolloids interact with starch through hydrogen bonding and electrostatic forces, increasing viscoelasticity, immobilizing water, inhibiting retrogradation, and physically shielding starch granules from enzymatic accessibility, thereby reducing digestibility and glycemic response [171,172]. Concurrently, NSPs form electrostatically driven complexes with rice glutelin, stabilizing protein conformation and reinforcing matrix integrity [173]. NSPs–lipid interactions further enhance matrix compactness by promoting hydrogen-bonded networks, influencing amylose–lipid V-type complex formation, and improving mechanical and barrier properties during thermal and extrusion processing [151,174]. By simultaneously reorganizing starch hydration, protein conformation, lipid association, and water distribution, NSPs generate a dense, reinforced composite matrix that acts as both a physical barrier and a chemically interactive environment for phenolic compounds. This integrated matrix protects phenolics during processing and early digestion and also synchronizes their release with NSPs degradation during colonic fermentation, effectively coupling phenolic bioaccessibility to controlled matrix digestion.

The strength and mode of NSPs–phenolic interactions are highly sensitive to polysaccharide molecular architecture, enabling tunable control over phenolic binding and stability. Mechanistic studies demonstrated that modifying NSPs structure directly alters phenolic complexation behavior. For example, ultrasound-modified pectin exhibits altered conformation and charge distribution, leading to distinct binding mechanisms and enhanced stability of C3G [175]. Structure–function analyses of arabinoxylans further revealed that substitution patterns, extractability, and feruloylation degree govern viscosity and physiological performance [176]. Additional evidence from polysaccharide network systems incorporating anthocyanins or polyphenol nanocomplexes confirms that polymer matrices stabilize phenolics and control functional outputs, as validated by FTIR and performance assays [177,178]. These findings demonstrate that NSPs regulate phenolic behavior through network entrapment, hydrogen bonding and electrostatic interactions, and microenvironmental control of diffusion and stability [179]. Recognizing NSP-driven integration of starch, protein, and lipid interactions as the dominant mechanism governing phenolic binding provides a unifying framework for rice phenolic functionality and supports matrix-oriented strategies for designing rice-based foods with enhanced phenolic retention, targeted colonic release, and metabolic benefits.

## 7. Role of Rice Lipids in Phenolic Binding and Matrix Function

Rice lipids play a multifunctional role in phenolic binding by directly contributing phenolic–lipid conjugates and by creating hydrophobic microenvironments that stabilize phenolics and modulate their bioavailability within the rice matrix. Lipid fractions in rice, particularly rice bran oil, are enriched with ferulate-derived compounds and phytosterol ferulates, underscoring the technological and nutritional relevance of lipid-associated phenolics [3]. Molecular studies demonstrated that esterification of phenolics with lipid moieties, such as phytosterols, alters functional performance and distinguishes the behavior of lipid-bound ferulates from that of free phenolic acids [180]. Beyond starch interactions, lipid-associated phenolics and lipid-mediated matrix reorganization collectively enhance phenolic stability across bran-rich, polished, cooked, and stored rice systems, shaping functional outputs such as antioxidant retention and glycemic modulation [181]. By integrating phenolic conjugation, starch reordering, and hydrophobic domain formation, rice lipids emerge as critical modulators of phenolic stability and delivery, providing a mechanistic foundation for lipid-based strategies, including self-emulsifying systems, to enhance phenolic efficacy in functional rice foods.

## 8. Rice Matrix Interactions with Intrinsic and Exogenous Phenolics

**Figure 4:**
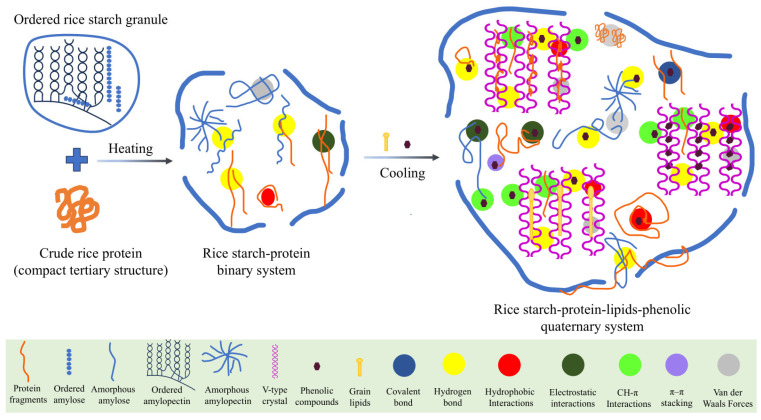
Schematic illustration of the proposed intermolecular interactions in gelatinized rice matrix depicting the coordinated interactions among primary components such as amylose, amylopectin, proteins, lipids, and phenolics. The illustration emphasizes the formation of multiscale networks, particularly through V-type inclusion complexes and phenolic binding to starch and protein.

In rice, phenolic functionality is governed by its integration within the native grain matrix, where interactions with starch, proteins, lipids, and dietary fibers collectively regulate starch digestibility, phenolic stability, and bioaccessibility. The proposed intermolecular interaction mechanisms of rice matrix and phenolics are depicted in Figure 4. Experimental studies consistently showed that the presence of rice proteins, dietary fiber fractions, and lipids significantly reduces the rate and extent of starch hydrolysis compared with purified starch systems [136,182]. Beyond cell wall polysaccharides covalent binding, intrinsic rice phenolics also engage in secondary non-covalent interactions with starch and rice proteins, forming compact macromolecular assemblies that further restrict diffusion and enzymatic access [14,16,79,183,184]. Processing-induced events such as starch gelatinization, amylose leaching and aggregation, and protein unfolding can partially reorganize these interactions by exposing new binding domains; however, phenolic release remains limited unless the NSPs network is disrupted [16,184,185,186,187]. Further, thermal treatments can promote the liberation of matrix-bound phenolics while simultaneously triggering partial degradation, resulting in a dynamic balance between phenolic release and thermal instability [5,6,14]. These intrinsic matrix–phenolic interactions function as a controlled retention system that synchronizes phenolic release with colonic metabolism, underpinning the gut health benefits associated with whole-grain and pigmented rice consumption.

Phenolics introduced through fortification or processing interact with the rice matrix primarily via non-covalent mechanisms, enabling matrix-level modulation of starch digestibility, phenolic bioaccessibility, and functional performance. Exogenous phenolics associate with gelatinized rice starch and proteins through covalent bonding, hydrogen bonding, hydrophobic interactions, van der Waals forces, CH–π interactions, and π–π stacking, forming starch–phenolic and protein–phenolic complexes [5,16,188]. Processing strategies such as hydrothermal treatment, extrusion, and product reformulation enhance these interactions by increasing molecular mobility and exposing binding sites, leading to pronounced reorganization of starch crystallinity and network architecture [16,23,189].

During the processing of rice grains, numerous phenolic interaction mechanisms mediated by matrix components are observed. The accumulated evidence suggests a key mechanistic pathway involves the formation of V-type amylose inclusion complexes, in which specific phenolic molecules associate with single-helical amylose structures to generate highly ordered assemblies classified as RS5 [16,23,189]. These complexes restrict starch swelling, reduce enzymatic accessibility, and enhance resistance to amylolysis [190]. Simultaneously, protein–phenolic interactions, particularly with rice glutelin, induce conformational rearrangements such as increased β-sheet content and compact aggregation, stabilizing phenolics against degradation while modifying protein digestibility [145]. Although exogenous phenolics are generally more bioaccessible than intrinsic phenolics, their release remains governed by diffusion-limited transport and adsorption within starch–protein networks, especially during rehydration and cooking [191,192]. Collectively, these matrix-mediated interactions provide a mechanistic foundation for designing phenolic-enriched, low-glycemic rice foods with enhanced antioxidant stability and metabolic benefits.

## 9. Conclusions and Future Perspectives

This review consolidates existing knowledge on rice phenolics and their interactions within the rice matrix, highlighting their role as a central determinant of rice’s nutritional value, functionality, and processing performance. Although rice contains a wide array of phenolic compounds, their biological significance is ultimately dictated not by their concentration alone but by their integration within the multicomponent rice matrix during processing. Across interaction levels, phenolic binding induces coordinated multiscale structural reorganization, encompassing amylose helix inclusion and V-type crystallinity, protein conformational rearrangements, lipid-assisted inclusion complex formation, and matrix compaction mediated by dietary fiber networks. These structural effects translate into consistent functional outcomes, including enhanced phenolic stability, reduced starch digestibility and glycemic responses, increased resistant starch formation, and improved antioxidant and technofunctional performance. Collectively, this body of evidence propose rice matrix–phenolic interactions as a crucial factor influencing the nutritional quality and metabolic functionality of both pigmented and non-pigmented rice products.

Future research should advance beyond reductionist binary systems toward integrated whole-matrix and food product models that capture the spatial, compositional, and dynamic complexity of real rice foods. Priority should be given to combining molecular docking, advanced spectroscopy, chemometrics, and multiscale modeling to elucidate binding specificity and competitive interactions between phenolics and rice macromolecules. Greater emphasis is also needed on tracking the transformation and release of matrix–phenolic complexes during gastrointestinal digestion and colonic fermentation, including their interactions with gut microbiota. Standardized quantitative frameworks linking interaction strength, structural reorganization, and physiological outcomes, particularly glycemic modulation and phenolic bioaccessibility are essential. Ultimately, translating mechanistic insights into structure-guided processing, targeted fortification, and rational formulation strategies will enable the development of next-generation functional rice products aligned with metabolic health promotion and chronic disease prevention.

## Figures and Tables

**Table 2:** Summary of research studies investigating the interactions between rice starch and phenolic compounds.

Phenolic Type	Starch State	Modification Method	Interaction Mechanism	Structural Evidence	Functional Outcome	Reference
Ferulic acid, gallic acid, *p*-coumaric acid, and p-hydroxybenzoic acid	Native	Mixing and Hydrothermal (95 °C)	Amorphous binding Non-inclusion complex: H-bonding V-type inclusion complexation (phenolic acid-specific)	Phenolic acid-specific: ↑ V-type crystalline peaks, ↓ relative crystallinity, ↓ short-range order, ↓ change in enthalpy, ↑ mean particle size, ↑ zeta potential, ↓ λ _max_.	↓ RDS, ↑ RS, ↓ starch digestibility, ↓ retrogradation enthalpy.	[84]
Caffeic acid, ferulic acid, epigallocatechin gallate, and tannic acid	Native	Extrusion	Non-inclusion complex: H-bonding V-type inclusion complexation	↓ relative crystallinity, ↑ short-range molecular order, double helix, ↓ molecular weight, ↑ Volume Weighted Mean, ↓ α-1,6 linkages glycosidic bonds, ↑ α-1,4 linkages glycosidic bonds.	↓ water absorption, ↑ water solubility, ↓ water holding capacity, ↓ Peak, breakdown, setback viscosity, ↓ Gel moduli (G′/G′′).	[85]
Ferulic acid, and gallic acid	Native	Mixing and Hydrothermal (95 and 60 °C)	Single-helix complex	↓ pH, ↓ relative crystallinity, ↓ double helix structure.	↓ RDS, ↑ RS/SDS, ↑ phenolic bioaccessibility and antioxidant activity, ↓ viscosity, ↓ 3D printing accuracy, ↓ Gel moduli (G′/G′′).	[86]
16 phenolic acids	Native	Heating with 50% (*v*/*v*) ethanol solution	Non-inclusion complex: H-bonding, hydrophobic interaction V-type inclusion complexation (structure-specific)	↑ V-type peaks, ↑ relative crystallinity, ↓ degree of short-range molecular order.	↑ RS, ↓ predicted glycemic index, ↓ Gel moduli (G′/G″).	[87]
16 phenolic acids	Debranched and gelatinized	Debranching and complexation	Non-inclusion complex: H-bonding, hydrophobic interaction V-type inclusion complexation (structure-specific)	↑ V-type peaks, ↑ relative crystallinity, ↓ degree of short-range molecular order, ↓ λ _max_.	↑ RS, ↓ RDS, ↑ breakdown viscosity, ↑ water solubility index, ↑ water holding capacity, ↓ swelling power, ↓ predicted glycemic index, ↓ syneresis rate, ↑ peak viscosity.	[88]
Chlorogenic acid	Native	Ultrasound and HPH	Non-inclusion complex: H-bonding V-type inclusion complexation	↑ V-type peaks, ↓ relative crystallinity, ↑ Volume Weighted Mean, Surface Area Weighted Mean, ↓ derivative weight loss.	↓ RDS, ↑RS/SDS, ↓ starch hydrolysis index, ↓ viscosity.	[89]
Black and brown rice bran phenolics	Native	Mixing	Non-inclusion complex: H-bonding V-type inclusion complexation	↑ 13.1° peak, ↓ degree of short-range molecular order, ↓ change in enthalpy, ↓ relative crystallinity.	↓ retrogradation enthalpy, ratio of the retrogradation, ↓ viscosity.	[90]
Tannic acid	Native	Ultrasound	Non-inclusion complex: H-bonding V-type inclusion complexation	↑ V-type peaks, ↓ λ _max_, ↓ relative crystallinity, ↓ degree of double helix, ↓ derivative weight loss.	↓ RDS/SDS, ↑ RS, ↑ Gel moduli (G′/G″).	[91]
Gallic acid, and sinapic acid	Distilled water or PAW gelatinized	Hydrothermal and ultrasound	V-type inclusion complexation	↑ V-type peaks, ↑ H^1^-NMR spectra (4.4–5.6 ppm), FTIR: new peaks at 1685 and 1447 cm^−1^, ↑ change in enthalpy.	↑ RS, ↓ water absorption, ↑ oil absorption capacity, ↓ water solubility index, ↓ swelling power, ↓ Gel moduli (G′/G″), ↑ DPPH antioxidant activity.	[92]
Ferulic acid, gallic acid, and quercetin	Native	Hydrothermal (95 °C), retrogradation	Non-inclusion complex: H-bonding GA: V-type inclusion complexation	↓ degree of double helix, ↓ change in enthalpy. GA: ↑ V-type peaks, FTIR: new peaks at 1685 and 1447 cm^−1^.	FA and GA: ↑ water solubility index, ↓ swelling power, ↓ peak viscosity, GA: ↓ retrogradation enthalpy.	[93]
Gallic acid	Native	HPH, Hydrothermal (95 °C)	Non-inclusion complex	↑ degree of short-range molecular order, ↑ fluorescence intensity.	↑ RS, ↓ predicted glycemic index, ↑ viscosity, ↑ Gel moduli (G′/G″).	[94]
Caffeic acid	Native	hydrothermal treatment	Non-inclusion complex: H-bonding, Van der Waals forces	↓ change in enthalpy.	↓ retrogradation enthalpy, ratio of the retrogradation.	[95]
(+)-catechin	Native	Mixing	Non-inclusion complex: H-bonding	↔ change in enthalpy.	↓ retrogradation enthalpy, ratio of the retrogradation, ↓ viscosity, ↑ gel elasticity, ↓ Gel moduli (G′/G″), ↓ peak viscosity.	[96]

↑, increased; ↓, decreased; ↔, no change; GA, gallic acid; FA, ferulic acid; PAW, plasma-activated water; HPH, high-pressure homogenization; RDS, rapidly digestible starch; SDS, slowly digestible starch; RS, resistant starch.

**Table 3:** Summary of research studies investigating the interactions between rice protein and phenolic compounds.

Phenolic Type	Protein System	Interaction Mechanism	Structural Evidence	Functional Outcome	Reference
Ferulic acid, gallic acid, and tannic acid	Rice protein	Non-covalent interactions: H-bonding, hydrophobic forces	↑ ζ-potential, ↓ sulfhydryl group, ↓ α-helix and β-sheet, ↑ β-turn and random coil, ↑ fluorescence red-shift, ↓ surface hydrophobicity.	↑ antioxidant activity, ↑ emulsion oxidative stability, ↑ inhibiting lipid oxidation, ↑ emulsion rheology and interfacial structure.	[113]
Purple rice native C3G and phenolics	Purple rice protein alkaline and enzymatic hydrolysate	Covalent bonds Non-covalent interactions: hydrogen bonding, hydrophobic interactions, π–π stacking	↑ α-helix and β-sheet, ↓ random coil structure, ↑ fluorescence intensity, ↑ hydrophobicity, ↑ sulfhydryl groups.	Alkaline hydrolysate: ↑ thermal stability, ↓ water and oil absorption capabilities, ↑ foaming and emulsifying properties.	[114]
Ferulic acid, gallic acid, and tannic acid	Rice protein	Covalent bonds Non-covalent interactions	Covalent complexes: ↑ polyphenol grafting, ↓ free amino/thiol groups, ↓ surface hydrophobicity, ↓ α-helix and β-sheet, ↑ random coil, ↑ ζ-potential, ↑ solubility. Non-covalent, ↓ β-turn and random coil.	Covalent complexes: ↑ emulsifying stability and activity index, ↑ antioxidant activity, ↓ phenolic bioaccessibility Non-covalent: ↑ phenolic bioaccessibility.	[115]
(+)-catechin	Rice bran protein isolate	Covalent bonds	↑ ζ-potential, ↓ particle size, ↓ α-helix and β-sheet, ↑ β-turn and random coil, ↑ electrostatic repulsion.	↑ emulsion stability, ↑ viscosity and shear stress, ↑ oxidative stability.	[116]
Ferulic acid (free and laccase-oxidized derivatives)	Rice protein isolate	Non-covalent interactions: hydrophobic interactions Covalent bonds: laccase-mediated quinone linkage	NR	↑ phenolic bioaccessibility, ↑ antioxidant activity, ↔ permeability coefficient	[15]
Ferulic acid	Rice bran protein hydrolysates pretreated with HHP	Covalent bonds: quinone addition	↑ α-helix, ↓ β-sheet, β-turn, and random coil, ↑ surface hydrophobicity, ↓ fluorescence intensity, ↑ turbidity, ↑ ζ- potential, ↑ particle size.	↑ antioxidant activity, ↑ emulsifying activity and emulsion stability	[117]
Black rice anthocyanins extract	Isolated rice protein and its constituent fractions	Non-covalent interactions: H-bonding, hydrophobic	↑ β-sheet, ↓ α-helix, ↑ amide II shift, ↑ fluorescence quenching.	↑ antioxidant activity, ↑ Foaming capacity and foaming stability	[118]
Epigallocatechin gallate	Rice glutelin	Non-covalent interactions: H-bonding, van der Waals forces	↓ α-helix, ↑ β-sheet, ↑ random coil structure, ↓ surface hydrophobicity.	↑ antioxidant activity.	[119]
Procyanidin dimer (PB2)	Rice glutelin	Non-covalent interactions: hydrophobic interactions	↓ α-helix, ↓ random coil structure, ↓ surface hydrophobicity.	↑ antioxidant activity, ↔ emulsification.	[120]
Chlorogenic acid	Rice protein trypsin hydrolysates	Covalent bonds	↑ droplet size, ↑ ζ- potential, ↑ fluorescence red shift, ↑ amide I and II shifts.	↑ emulsifying activity, ↑ oxidative stability, ↑ lipid oxidation inhibitions, ↑ interfacial film.	[121]
Ferulic acid	rice protein isolate	Non-covalent interactions: hydrophobic interactions	↓ β-turn, ↓α-helix, ↑ random coil, ↑ β-sheet, ↓ amide I band and amide II band.	↑ emulsion oxidative stability, ↑ antioxidant activity.	[122]
Epigallocatechin gallate	Rice bran albumin	Non-covalent interactions: H-bonding, van der Waals forces	↑ Amide I shift, ↑ aggregation.	↑ thermal stability, ↓ antioxidant activity.	[123]
Tea catechins	Rice bran protein isolate	Non-covalent interactions: H-bonding, van der Waals forces, hydrophobic interactions	↑ random coil, ↑ α-helix, ↑ fluorescence quenching.	↑ intestinal recovery.	[124]
Gallic acid	Rice glutelin	Non-covalent interactions: H-bonding, van der Waals forces	↓ α-helix, ↑ β-sheet, ↑ fluorescence quenching, ↓ surface hydrophobicity.	NR	[125]

↑, increased; ↓, decreased; ↔, no change; HHP, high hydrostatic pressure; NR, not reported.

## Data Availability

No new data were created or analyzed in this study. Data sharing is not applicable to this article.

## Abbreviations

TPC, total phenolic contents; TAC, total anthocyanin contents; TFC, total flavonoid content; C3G, cyanidin-3-*O*-glucoside; DP, degree of polymerization; NSPs, Non-starch polysaccharides; RS5, resistant starch type 5.
